# Modular assembly of designer PUF proteins for specific post-transcriptional regulation of endogenous RNA

**DOI:** 10.1186/1754-1611-8-7

**Published:** 2014-03-01

**Authors:** Zhanar Abil, Carl A Denard, Huimin Zhao

**Affiliations:** 1 Department of Biochemistry, University of Illinois at Urbana-Champaign, Urbana, IL 61801, USA; 2 Department of Chemical and Biomolecular Engineering, University of Illinois at Urbana-Champaign, Urbana, IL 61801, USA; 3 Department of Bioengineering, Department of Chemistry, Center for Biophysics and Computational Biology, and Institute for Genomic Biology, University of Illinois at Urbana-Champaign, Urbana, IL 61801, USA

**Keywords:** Protein engineering, RNA-binding protein, Post-transcriptional regulation, PUF, Pumilio, Tristetraprolin, TTP, Golden Gate

## Abstract

**Background:**

Due to their modular repeat structure, Pumilio/fem-3 mRNA binding factor (PUF) proteins are promising candidates for designer RNA-binding protein (RBP) engineering. To further facilitate the application of the PUF domain for the sequence-specific RBP engineering, a rapid cloning approach is desirable that would allow efficient introduction of multiple key amino acid mutations in the protein. Here, we report the implementation of the Golden Gate cloning method for an efficient one-step assembly of a designer PUF domain for RNA specificity engineering.

**Results:**

We created a repeat module library that is potentially capable of generating a PUF domain with any desired specificity. PUF domains with multiple repeat modifications for the recognition of altered RNA targets were obtained in a one-step assembly reaction, which was found to be highly efficient. The new PUF variants exhibited high *in vitro* binding efficiencies to cognate RNA sequences, corroborating the applicability of the modular approach for PUF engineering. To demonstrate the application of the PUF domain assembly method for RBP engineering, we fused the PUF domain to a post-transcriptional regulator and observed a sequence-specific reporter and endogenous gene repression in human cell lines.

**Conclusions:**

The Golden Gate based cloning approach thus should allow greater flexibility and speed in implementing the PUF protein scaffold for engineering designer RBPs, and facilitate its use as a tool in basic and applied biology and medicine.

## Background

The presence of RNA-binding proteins (RBPs) in RNA biology is ubiquitous. Hundreds to thousands of eukaryotic proteins are estimated to function as RBPs [1] and govern many aspects of RNA biology including translation, turnover, processing, and cellular localization [2-4]. Despite their great diversity in function, only a few types of RNA-binding domains are known, which are combined in different structural arrangements with a variety of functional domains [5]. This modular architecture makes RBPs an attractive tool for studying the vast complexity of eukaryotic transcriptomes as well as manipulating RNA for therapeutic purposes [6,7].

The function of many RBPs can be studied [8-11] by tethering them to a reporter RNA through a well-characterized RNA-binding peptide with a fixed specificity [12]. However, this approach can only be applied to manipulate heterologous RNA because prior tagging of the RNA is required. In order to manipulate endogenous RNA in its native expression conditions, one could envision a designer RBP with an RNA-binding scaffold that could be easily engineered for sequence specificity. To date, only pentatricopeptide repeat [13,14] and Pumilio/fem-3 mRNA binding factor (PUF) [15-18] proteins have been demonstrated to have the potential to be rationally modified for predictable and specific RNA recognition.

**Figure 1:**
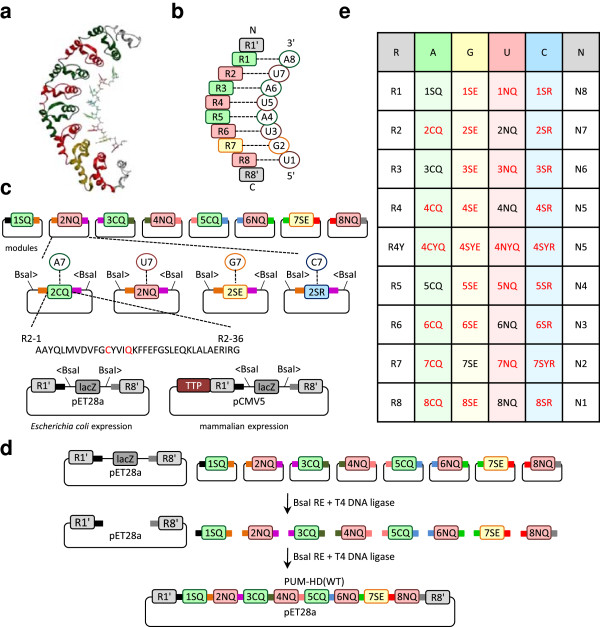
**The GG library and assembly schematic. (a)** Crystal structure of PUM-HD bound to RNA, adapted from reference [15], GenBank ID code 1M8Y. **(b)** Schematic of PUM-HD bound to RNA. Filled boxes, PUF modules. Circles, RNA bases. **(c)** Schematic of the main library components: 8 repeat modules, with matching overhangs colored in identical colors; 4 variations of module 2, with corresponding recognition nucleotide indicated above; the aa sequence of module 2, with mutant aa indicated in red; and two receiving vectors. **(d)** The GG assembly schematic. A one-pot reaction that contains 8 modules of choice, a receiving vector, and enzymes allows the creation of 9 unique overhangs. The exact matching of the overhangs results in the predetermined repeat order assembled in the receiving vector. **(e)** Schematic of the GG library. R, module; N, nucleotide; recognized nucleotides indicated in the top row. First and last letters in the module names represent aa residues 12 and 16, in each module, respectively. Middle letter, if present, represents the “stacking” aa 13. Black font, WT modules. Red font, mutant modules. Green, yellow, pink, and blue fillings for modules recognizing A, G, U, and C, respectively.

PUF proteins are eukaryotic RBPs that are involved in post-transcriptional gene regulation [19]. The crystal structure of Pumilio homology domain (PUM-HD), the RNA-binding region of the human Pumilio 1 (PUM1) protein (Figure 1a), reveals 8 structural repeats, each containing ~36 amino acids (aa), and flanking N-and C-terminal regions [20,21]. The structure also suggests that recognition of the target RNA sequence is highly modular since each repeat binds to a single RNA base [15]. The N-terminal repeat (R1) binds to the 3′-nucleotide residue (N8) of the target sequence (Figure 1a and b), while the C-terminal repeat (R8) binds to the 5′-nucleotide residue (N1). Residues at positions 12 and 16 in each repeat directly interact with a Watson-Crick edge of a base, whereas the residue at position 13 is involved in a stacking interaction between two adjacent bases [15]. The structure suggests a recognition “code”, where residues at positions 12 and 16 in each repeat contribute to specific recognition of a base, with N_12_Q_16_ recognizing uracil, C_12_Q_16_ adenine, and S_12_E_16_ guanine [15]. The residue combination S_12_R_16_ was engineered to recognize cytosine [17,18]. By swapping the key residues at these positions, it was shown that designed PUF proteins with altered specificity could be engineered [16,22]. In the past several years, engineered PUF domains were successfully fused to different effector domains for polyadenylation of an endogenous gene or repression of a reporter gene in *Xenopus*[23], cleavage of a mitochondrial-encoded gene in human cells [24], splicing regulation of endogenous *Bcl*-*X* pre-mRNA in human cells [25], and imaging endogenous RNA [26-28]. These advancements demonstrate the growing potential for the RBPs with various functional domains and engineered specificity.

However, PUF-based RBPs are still far from widespread implementation. One of the limitations in engineering PUF domains with novel specificities is the lack of a cloning platform capable of rapid and efficient introduction of multiple mutations in separate repeats simultaneously. In this study, we report the implementation of the Golden Gate (GG) cloning, Type IIS restriction endonuclease-based approach [29] for engineering of PUF-based RBPs. To demonstrate the efficiency of this approach, we first used this cloning method for construction of several mutant PUF domains with novel specificities and assayed their binding affinities. Second, we linked the RNA-binding activity of engineered PUF domains to the translational repression activity of tristetraprolin (TTP), and used the fusion protein in a functional reporter system to assay the PUF domain activity in HeLa cells. Finally, we showed the application of the engineered TTP-PUF fusion proteins for the post-transcriptional regulation of an endogenous gene in HEK293 cells.

## Results and discussion

### Efficient assembly of a custom-designed PUF domain

The GG cloning method, which is implemented here for the assembly of custom PUF domains, is based on the ability of Type IIS restriction enzymes to cleave outside of their non-palindromic recognition sequence [29], thus creating overhangs unrelated to the recognition sequence. This polarity and flexibility in the overhang sequence allows for a seamless removal of the original restriction site as well as a ligation of multiple fragments in one step.

As a scaffold for the development of the assembly toolkit, we used the human PUM-HD, which consists of the amino acids 828-1176 of the full-length PUM1 [21]. Each of the 8 structural repeats of PUM-HD was cloned individually into a pNEB193-based “intermediate vector” and was used as a separate assembly module (Figure 1c). We designed all 8 modules as well as the *lacZ*α gene in the “receiving vector” to be flanked by two BsaI sites in such a way that would allow creation of 9 unique overhangs (Figure 1c). In a one-pot reaction, the 8 modules and the receiving vector can be efficiently cut and re-ligated in a predefined order (Figure 1d).

These 8 modules were further expanded into a library where each module has 4 variations for the recognition of any of the 4 nucleotides (nt), consistent with the PUF recognition “code” [15,17,18]. The variants of the same module position have the same overhangs and the same amino acids as the wild type (WT) module except at positions 12 and 16 (Figure 1c). The introduced mutations are uniform across modules (Figure 1e), except for module 7 for the recognition of cytosine, where the “stacking” residue was also substituted with tyrosine, in accordance with a previous report by Dong *et al*. [18]. Since module 3S_12_R_16_ requires a tyrosine as a “stacking” residue in the adjacent module 4 for *in vivo* activity [18], four additional module 4 variants were created, where the “stacking” residue at position 13 was mutated from histidine to tyrosine.

Finally, we constructed two receiving vectors, pET28-GG-PUF for *Escherichia coli* expression and pCMV-TTP (C147R)-GG-PUF for mammalian expression. They both contain a *lacZ*α gene for easy identification of assembled clones using blue-white screening, as well as the flanking N- and C-terminal regions of PUM-HD, which we refer to as R1′ and R8′, respectively. The entire library hence consists of 36 intermediate vectors and 2 receiving vectors. Thus, our library of PUF repeat modules is potentially capable of a one-step assembly of PUF domains with specificity for any RNA sequence of 8 nt, given that they can be expressed in a soluble manner.

In order to test the efficiency of the method, we re-assembled the original PUM-HD from the WT modules into both receiving vectors. We analyzed 10 randomly chosen clones from each assembly by restriction digestion and found that all produced the anticipated digestion pattern (Additional file 1: Figure S1a and b). Next, we sequenced one clone from each assembly and found that both sequences were correct, indicating that the assembly process is highly efficient regardless of a receiving vector. Assembly of the consecutive mutant PUF domains was as efficient as the assembly of the WT PUF domain (data not shown). The entire process takes 3 days, with the GG reaction and *E. coli* transformation on day 1, colony picking on day 2, and plasmid purification and digestion-confirmation of the clones on day 3. The procedure is therefore ideal for the rapid introduction of multiple mutations in a PUF domain with high efficiency.

### Binding activity of custom-designed PUF domains *in vitro*

With the combinatorial assembly tool in hand, we set to determine if increasing the number of mutations affects the activity or specificity of a PUF domain. We assembled four variant PUF domains with 2, 4, 6, and 8 mutant modules that were named PUF (S2), PUF (S4), PUF (S6), and PUF (S8), respectively (Table 1). To test their *in vitro* activity, we assayed the binding affinity of the proteins to WT and their cognate RNA sequences using a fluorescence polarization assay (Additional file 1: Figure S2b-f). We found that the WT PUF as well as the PUF variants all bound to their cognate RNA sequences with high affinity (Table 1). As predicted, all of the PUF proteins exhibited lower affinity to noncognate RNA, which contained between 2 and 8 nucleotide mismatches with the cognate RNA (Table 1, Additional file 1: Figure S2b-f). The binding affinities to cognate sequences decreased from PUF (WT) to PUF (S8), indicating that there is a weak negative correlation between the number of mutations and binding affinity to cognate RNA, although the correlation is not strictly linear (Table 1). However, even PUF (S8), which has 8/8 mutant modules, binds specifically and with high affinity to its cognate RNA. These results further corroborate the study by Cheong and Hall, who demonstrated the specificity and modularity of PUF protein target recognition [16].

**Table 1:** **Mutations**, **cognate and noncognate RNA oligonucleotide sequences**, **and binding affinities of PUF** (**WT**), **and PUFs S2**-**S8**

**Protein**	**Protein modules**	**Cognate RNA sequence**	**K**_**D**_, **nM**	**Noncognate RNA sequence**	**K**_**D**_, **nM**
	**Ct 8 – 7 – 6 – 5 – 4 – 3 – 2 – 1 Nt**	**5**′ **1 2 3 4 5 6 7 8 3**′		**5**′ **1 2 3 4 5 6 7 8 3**′	
PUF(WT)	NQ-SE-NQ-CQ-NQ-CQ-NQ-SQ	CCAGAAU/UGUAUAUA/UUCG	0.043±0.023	CCAGAAU/**A**GUAUAU**U/**AUUCG	3.56±0.90
PUF (S2)	**CQ**-SE-NQ-CQ-NQ-CQ-NQ-**NQ**	CCAGAAU/**A**GUAUAU**U/**AUUCG	0.76±0.11	CCAGAAU/UGUAUAUA/UUCG	6.29±2.80
PUF (S4)	NQ-SE-**CQ**-**NQ**-**CYQ**-**NQ**-NQ-SQ	CCAGAAU/UG**AUAU**UA/UUCG	0.59±0.10	CCAGAAU/UGUAUAUA/UUCG	5.77±1.33
PUF (S6)	**CQ**-SE-**CQ**-**NQ**-**CYQ**-**NQ**-NQ-**NQ**	CCAGAAU/**A**G**AUAU**U**U/**UUCG	6.05±0.25	CCAGAAU/UGUAUAUA/UUCG	89.6±16.5
PUF (S8)	**CQ**-**NQ**-**CQ**-**NQ**-**CYQ**-**NQ**-**SE-NQ**	CCAGAAU/**AUAUAUGU/**UUCG	2.79±0.69	CCAGAAU/UGUAUAUA/UUCG	ND

Bold, mutant modules and changed RNA bases compared with the WT target sequence. Target RNA sequences are delimitated by forward slashes. Ct, C-terminus, and Nt*,* N-terminus of the protein. K_D_ values were determined using nonlinear curve fitting and represent the mean ± SD (n = 3). *ND*, K_D_ not determined due to little binding at 300 nM PUF (S8) to WT RNA.

### Engineering and assessment of designer repressor-RBP, TPUF

As an example of PUF-based RBP engineering, we fused the PUM-HD domain to TTP, a well-studied post-transcriptional regulator. TTP binds to AU-rich elements (AREs) in the 3′-untranslated region (UTR) of the target genes and promotes mRNA degradation by recruiting a deadenylase complex [30]. TTP is also known to promote ARE-dependent gene knockdown via translation repression through cooperation with a general translation inhibitor RCK/P54 [31], though the details of this mechanism remain unknown. We reasoned that the RNA-binding activity of TTP was undesirable due to possible interference of TTP towards efficient binding of PUF to PUF-binding sites (PBSs). We therefore introduced the C147R mutation that was shown to abolish the binding of TTP to ARE [32]. TTP (C147R) was fused to the N-terminus of PUM-HD through a (G_4_S)_3_G_4_ linker and expressed in HeLa cells for functional analysis (Additional file 1: Figure S3a).

**Figure 2:**
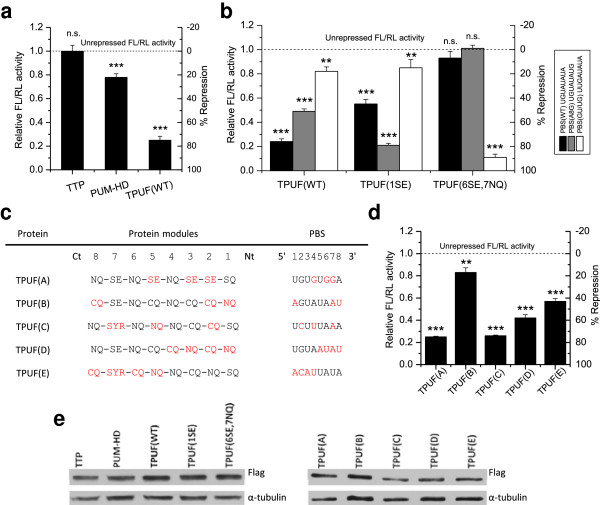
**Various TPUF repression activity assessment in HeLa cell line. (a)** Dual luciferase assay shows that TPUF (WT), a fusion of TTP (C147R) and PUM-HD (WT), exhibits the greatest down-regulation activity on FL_PBS (WT)_ expression, compared with TTP and PUM-HD (WT) alone. Data represented as mean fold change relative to cells transfected with FL_Random_ (dashed line, unrepressed FL/RL activity) ± SD: n.s., not significant, ***P ≤ 0.001 (n = 3, t test). **(b)** Luciferase assay shows predicted specificity of previously reported PUF mutants [16]. TPUF (WT) prefers PBS (WT), TPUF (1SE) prefers PBS (A8G), and TPUF (6SE,7NQ) prefers PBS (GU/UG). Data represent means ± SD: n.s., not significant, **P ≤ 0.01, ***P ≤ 0.001 (n = 3, t test). **(c)** Mutations and PBSs of TPUFs A-E with 3-4 randomly chosen mutant modules. Black, WT PUF modules and corresponding RNA bases. Red, mutant PUF modules and corresponding RNA bases. Ct, C-terminus, Nt, N-terminus of the protein. **(d)** A graph of luciferase activity, where TPUFs A-E repress FLs with cognate PBSs. Data represent means ± SD: **P ≤ 0.01, ***P ≤ 0.001 (n = 3, t test). **(e)** Western blot of effector proteins using anti-Flag antibody shows no major difference in the expression. Anti-α–tubulin antibody was used as a loading control.

To assay the gene repression activity of TTP-PUF fusion constructs, a dual luciferase assay was implemented. To the 3′ UTR of the firefly luciferase (FL) reporter, 10 PBSs separated by 6-18 nt were cloned (Additional file 1: Figure S3 b). *Renilla* luciferase (RL) lacking any PBSs was co-transfected with FL as a transfection control. FL_Random_, also lacking any PBSs, was used as a repression control. Values of FL_PBS_/RL normalized to FL_Random_/RL were reported as “relative FL/RL activity.” We observed that TTP (C147R) alone did not significantly repress the FL_PBS (WT)_ activity, whereas PUM-HD (WT) alone repressed FL_PBS (WT)_ by 20%. The observed weak activity of PUM-HD RNA-binding motif alone can be explained by previous findings that the Pumilio RNA-binding domain is also a translational regulator that is capable of recruiting deadenylases to the concave surface of repeats 7 and 8 [33]. However, the TTP (C147R)-PUM-HD (WT) fusion construct (hereafter referred to as TPUF (WT)) repressed the FL_PBS (WT)_ activity by 80% (Figure 2a), thus demonstrating that the fusion construct exhibits both specific RNA binding and high repression activity_._

We also tested the repression levels of FL containing 1, 3, and 5 PBSs in the 3′ UTR (Additional file 1: Figure S4) and observed repression activity of TPUF (WT) ranging between 31-55%. However, to obtain the greatest dynamic range of our reporter assay, we conducted consequent experiments using 10 PBSs. Although using 10 PBSs is in contrast compared with those using 1 PBS in previous assays [23], it is comparable to using 5-6 binding sites in tethering assays of TTP and other ARE-mediated decay activation domains [31,34,35].

To determine if the TPUF (WT) construct functions by promoting degradation of target RNA, we performed real-time PCR (RT-PCR) analysis on the FL reporter, and used RL as the internal control. We did not observe decrease in RNA levels in FL_PBS (WT)_ compared with FL_Random_ in the presence of TPUF (WT) (Additional file 1: Figure S5). Several FL RT-PCR primer pairs were used, and consistently no RNA destabilization was observed (data not shown). These results are in accordance with a similar TTP tethering assay [31], where luciferase activity was knocked down despite little RNA destabilization. We suggest that the TPUF constructs function similarly, by promoting translational repression rather than RNA degradation [31].

To further test the design concept, we used our GG toolkit to assemble two previously reported PUF variants [16]. TPUF (1SE) has repeat 1 replaced for recognition of G8 [PBS (A8G)], and TPUF (6SE,7NQ) has repeats 6 and 7 replaced for the recognition of G and U at positions 2 and 3, respectively [PBS (GU/UG)]. TPUF (WT) and the two PUF variants exhibited highest repression activities towards their cognate PBSs, with repression levels of 76%, 79%, and 88%, respectively. Compared with cognate PBSs, we observed a diminished activity towards PBSs with 1 or 2 mismatches, though some cross-reactivity is evident (Figure 2b). These observations are consistent with the cross-reactivity between WT and PUF (1SE) in *in vitro* assays [16] and similar cross-reactivity between WT and other mutant PUF proteins [16,25] that differ by 1-2 repeats. Overall, luciferase repression by the TPUF constructs was sequence-specific, corroborating the validity of the TPUF-reporter system.

**Figure 3:**
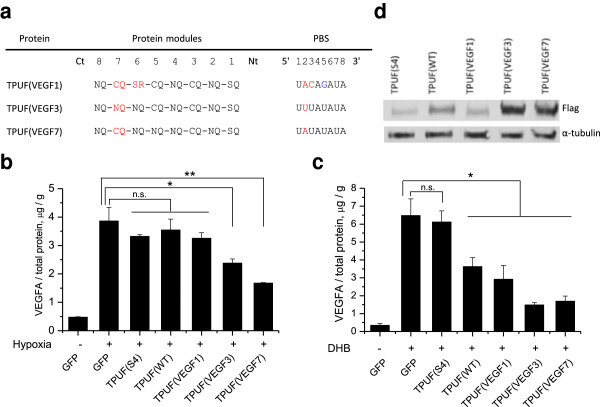
**TPUF represses endogenously expressed VEGFA gene in HEK293 cell line. (a)** Mutations and binding sequences of TPUFs designed for VEGFA 3′ UTR recognition. Black, WT modules and corresponding RNA bases. Red, mutant modules and corresponding RNA bases. Blue, a mismatch in the recognition sequence. Ct, C-terminus, Nt, N-terminus of the protein. **(b)** The graph demonstrates inhibition of hypoxia-induced VEGFA expression in cells transfected with engineered TPUFs VEGF3 and VEGF7. In hypoxic (+) cultures, VEGFA expression was induced with 500 μM CoCl_2_ 24 hours after transfection and then cultivated for 24 hours. Secreted VEGFA levels measured by ELISA were normalized to total protein amounts from lysed cells measured by Bradford Assay. Data represented as mean ± SD: n.s., not significant, *P ≤ 0.05, **P ≤ 0.01, (n = 3, t test). **(c)** The graph demonstrates inhibition of DHB-induced VEGFA expression in cells transfected with TPUFs WT, VEGF1, VEGF3, and VEGF7. HEK293 cells with the integrated V24P-GS60 transcriptional activator of endogenous VEGFA promoter were treated with 100 nM DHB 24 hours after TPUF transfection and then cultivated for 24 hours. ELISA and normalization to total protein amounts as in the previous panel. Data represented as mean ± SD: n.s., not significant, *P ≤ 0.05. **(d)** Western blot of effector proteins using anti-Flag antibody shows greater expression of TPUFs VEGF3 and VEGF7 compared with TPUF (WT) and other mutants. Anti-α–tubulin antibody was used as a loading control.

In order to further verify the functionality of the cloning method and the TPUF platform, we assembled more TPUF constructs with mutant repeats randomly introduced throughout the PUF domain (Figure 3c), denoted as TPUFs A-E. Out of 5 TPUF variants, only TPUF (B) showed low (17%) repression activity towards a cognate PBS, whereas TPUFs A, C-E showed repression activities ranging from 43% to 75% (Figure 3d). TPUFs with all 8 replaced modules demonstrated poor repression activities (data not shown), indicating that accumulation of mutations in the PUF domain does not always result in active TPUF proteins *in vivo*.

The difference between TPUF activities in a cell line could be dependent on the expression and solubility levels of the fusion proteins. We therefore investigated soluble expression levels of all of the effector proteins mentioned above using Western blotting (Figure 2e). We found that TTP, PUM-HD, and TPUF (WT), as well as mutants TPUF (1SE) and TPUF (6SE,7NQ) were expressed at similar levels, thus excluding the possibility of protein abundance variability distorting the observed repression activities. On the other hand, we noticed some noticeable variability among soluble expression levels of TPUFs (A-E). However, this variability does not seem to be consistent with the observed TPUF activities. In particular, it does not explain the apparent lower activities of TPUF (B) and TPUF (E).

We conclude that *in vivo* TPUF activities must be dependent on various other factors in addition to protein stability. For example, unequal contributions of different repeat-base interactions to the binding energy of the RNA-protein complexes [16,36] may result in different overall binding affinities to cognate RNA. In addition, binding to noncognate RNA [37] could allow sequestration of the protein to non-target RNA *in vivo*. Finally, sporadic domain interactions in fusion proteins could interfere with RNA binding. We cannot yet predict the contribution of each of these factors on PUF activity *in vivo*, and therefore suggest that the effects of these and other factors on engineered PUF activity have to be systematically investigated.

### Endogenous gene regulation using TPUF

As a proof of concept for implementing designer RBP for endogenous gene regulation, we designed TPUFs that bind to the 3′ UTR of human vascular endothelial growth factor-A (*VEGFA*) mRNA. VEGFA is one of the central mediators of angiogenesis, and was shown to be overexpressed in many human tumors. VEGFA is up-regulated in these tumors under hypoxic growth conditions that many tumors create [38]. As such, VEGFA is an attractive target for the development of therapeutics to inhibit pathological angiogenesis. We reasoned that for our TPUF repression assay, elevated VEGFA levels are more therapeutically relevant than physiological lower levels, and therefore employed two different strategies to up-regulate endogenous VEGFA expression. In the first strategy, we incubated HEK293 cells with μM 500 CoCl_2_, an agent that causes conditions mimicking hypoxia [39], and achieved an 8-fold VEGFA induction compared to cells grown in the absence of the agent (Figure 3b). In the second strategy, we used a HEK293 cell line in which a small molecule-responsive gene switch for VEGFA expression was stably integrated [40]. Upon induction with 4,4′-dihydroxybenzyl (DHB), endogenous VEGFA was up-regulated 19-fold.

For the post-transcriptional down-regulation of VEGFA, we assembled TPUFs VEGF1, VEGF3, and VEGF7 (Figure 3a), which have, respectively, 1, 3, and 7 PBSs in the 3′ UTR of all known transcript variants of *VEGFA* gene. PUF domains in the TPUF VEGF3 and VEGF7 constructs each carry one mutant repeat and were previously reported to be active *in vitro*[16]. TPUF (VEGF1) has repeats 6 and 7 replaced for the recognition of 2A and 3C in the PBS. WT repeat 4N_12_Q_16_ was left unchanged for the recognition of 5G (Figure 3a, blue), since this repeat is known to be promiscuous [41]. TPUF (S4) was shown to be active for its cognate RNA using the luciferase assay (Additional file 1: Figure S6); however, it does not have binding sites in the 3′UTR of *VEGFA* mRNA, and therefore was not expected to repress the gene. Fortuitously, PBS (WT) is present 3 times in the 3′ UTR of *VEGFA* mRNA, which allowed us to use TPUF (WT) as a reference for assaying activities of mutant TPUFs.

To assay the repression activity, we transiently transfected recombinant HEK293 cells with equimolar amounts of GFP or one of the TPUF constructs, induced the expression of VEGFA 24 hours after transfection, and assayed secreted levels of VEGFA by ELISA 24 hours after induction. VEGFA levels were normalized to total protein concentration, which we assumed to correlate with cell number. Bradford Assay was used to measure the total protein concentrations from cell lysates. We found that VEGFA amounted to less than 1% of total protein concentration by mass (Figure 3b and c), hence the variations of VEGFA concentrations in the cell would have no significant effect on total protein concentration. In both induction strategies, the VEGFA levels were not significantly affected by TPUF (S4) compared to samples transfected with GFP, as expected (Figure 3b and c). In cells treated with CoCl_2_, only TPUF (VEGF3) and TPUF (VEGF7) significantly repressed VEGFA expression, which was down-regulated by 38% and 57%, respectively (Figure 3b). On the other hand, in cells induced with DHB, TPUF (WT) caused considerable (44%) repression of VEGFA, whereas TPUFs VEGF1, VEGF3, and VEGF7 knocked down VEGFA levels by 55%, 77%, and 74%, respectively (Figure 3c). We are currently unaware of the reason why cells exposed to hypoxia were more resistant to VEGFA repression by our TPUF constructs compared with cells that expressed the gene switch. We speculate that it could be due to yet undiscovered transcriptional and/or translational gene regulatory response of cells to hypoxia. Increasing the TPUF expression time to 41 hours and decreasing exposure to hypoxia to 7 hours resulted in similar repression levels as in the previous conditions (data not shown), and therefore we exclude the possibility that this resistance is simply due to lower expression of TPUFs at hypoxia. The lower TPUF activity at hypoxia might hence be due to an unknown interference with TTP function. Nevertheless, we observed a considerable sequence-specific down-regulation of VEGFA in cells expressing the VEGFA transcriptional gene switch, and confirmed the efficacy of the TPUF platform in the down-regulation of an endogenous gene in human cells as yet another demonstration of the applicability of PUF-based RBPs at post-transcriptional gene regulation [23,24].

To compare the expression levels of TPUF constructs among each other, we performed a Western blot analysis of the effector proteins under normoxia (Figure 3d). We observed a substantially greater soluble expression of TPUF (VEGF3) and TPUF (VEGF7) which might be the main reason of these constructs’ higher overall activity.

These VEGFA repression levels are comparable to those in similar assays conducted with zinc finger transcriptional repressors or small interfering RNA (siRNA). For example, hypoxia-induced VEGFA protein levels in HEK293 cells were knocked down by 74% by an engineered zinc finger transcriptional repressor [42] and VEGFA mRNA was knocked down by 50% by another zinc finger repressor in HEK293 cells grown in normoxia [43]. On the other hand, siRNA knocked down endogenous VEGFA by up to 43% in ID8 cells [44] and up to 71% in hypoxic HEK293 cells [42]. Thus, the TPUF system that we have engineered is as effective as some other existing technologies that are often used to knock down gene expression levels, and can be a powerful alternative.

Based on these findings, we conclude that the implementation of TPUFs for down-regulation of other endogenous genes with high efficacy is possible. However, factors determining tight binding of Pumilio domains to RNA *in vitro*, as well as factors determining high *in vivo* activities are still largely unknown, and a systematic investigation is needed. For example, based on the results reported above, either effective target site recognition or protein stability may play a major role in a protein’s functional efficacy *in vivo* in any given mutant. It is difficult, at this stage, to predict which contribution would predominate. *In vitro* RNA binding affinities and *in vivo* activities show only a rough correlation [23,25], and soluble expression levels do not always predict activity differences (Figure 2d and e). Therefore, for engineering of TPUFs with novel specificities or PUM-HD-based proteins with novel functionalities, we recommend building a reporter system suitable for the given protein activity, and directly screening the functional efficacy of the assembled PUF variants.

## Conclusions

In this study, we established a toolkit for rapid engineering of designer RBPs that can be used for manipulation of endogenous genes. This approach should allow greater flexibility and speed at creating PUF domains with user-defined specificities and thus facilitate the use of PUF-based designer RBPs as a tool in research and therapeutics. We demonstrated that PUF mutants with as many as 8/8 mutant modules can be cloned with high efficiency and that the resulting proteins retain their specificity and high affinity to their cognate RNA *in vitro*. This result is consistent with the study by Cheong and Hall [16] and confirms the RNA recognition code and modularity of PUF domains. Finally, we were able to demonstrate the implementation of the protein-based post-transcriptional repressor for down-regulation of an endogenous gene. The TPUF platform introduced in this work exhibits modular and sequence-specific recognition and down-regulation of genes. We envision the future development of PUF-based RBPs with various functionalities that could range from endogenous RNA splicing, imaging, and localization to various base modifications and more. The development of rapid assembly tools for PUF specificity engineering, as presented in this work, could play a critical role in facilitating and enhancing these endeavors.

## Methods

### Materials

All the chemicals and solutions were purchased from Fisher Scientific (Pittsburgh, PA), unless noted otherwise. Oligonucleotides were purchased from Integrated DNA Technologies (Coralville, IA). All the enzymes were purchased from New England Biolabs (Ipswich, MA), unless noted otherwise.

### Library creation

The amino acid and DNA sequences of all the modules in our GG cloning library are listed in Additional file 1: Table S1. All the amplification primers used for the creation of the library are listed in Additional file 1: Table S2. The intermediate vector, pChlr-pNEB193, was created by replacing the original *amp* resistance gene in pNEB193 (New England Biolabs) plasmid with the *cam* resistance gene from pACYC (New England Biolabs) plasmid by Gibson Assembly (GA) [45]. Plasmid pTYB3-PUM1-HD [GenBank:D43951] was a gift of Dr. Traci M. Tanaka Hall (Addgene plasmid 17543). The WT GG assembly modules were amplified from pTYB3-PUM1-HD and inserted in the SacI and HindIII sites of the intermediate vector. The amplification primers also contained BsaI sites for subsequent GG cloning. Some of the modules’ 5′ and 3′ ends were modified with silent mutations (Additional file 1: Table S1) for the creation of non-overlapping BsaI overhangs. The mutant GG modules were created by GA from the corresponding WT modules.

The receiving vector pET28-GG-PUF was GA-cloned from the following fragments: pET28a (Novagen) digested with NdeI and SalI, flanking PUM-HD repeats R1′ and R8′ amplified from pTYB3-PUM1-HD, and *lacZ*α amplified from pNEB193. BsaI sites were introduced next to R1′ and R8′ for GG cloning of PUM-HD. The receiving vector pCMV-TTP (C147R)-GG-PUF was cloned in two steps. First, pCMV-TTP-GG-PUF was created by replacing PUM-HD with a *lacZ*α gene flanked by BsaI sites and removal of 3 existing BsaI sites in the pCMV-TTP (WT)-PUM-HD (see the effector plasmids section). Next, pCMV-TTP (C147R)-GG-PUF was GA-cloned by replacing Flag with 3xFlag and mutating the C147R of TTP in the plasmid pCMV-TTP-GG-PUF.

For the availability to the scientific community, we are making arrangements to deposit all the plasmids constituting the PUM-HD repeat library that we have developed here to the Addgene non-profit plasmid repository. These plasmids should be available to researchers within a month of the publication of this manuscript.

### Reporter plasmids

pCMV-Fluc plasmid was created by amplification of the firefly luciferase gene from pGL3 plasmid (Promega) and insertion into SacI and KpnI sites of pCMV5 vector (a gift of Dr. David Russell). All the pCMV-Fluc-10xPBS plasmids, as well as pCMV-Fluc-Random were cloned by primer-extension of 6 primers (Additional file 1: Table S3) carrying 10 PBSs and subsequent GA-cloning into PstI and XmaI sites of the pCMV-Fluc plasmid. The 6-18 nt spacers between the 10 PBS in the 3′ UTR of the FL were the same in different FL-PBS sequences, and were designed in such a way to minimize secondary structure formation that would involve these spacers. The pCMV-Fluc-Random was created by replacing all the PBSs in the pCMV-Fluc-10xPBS with 10 different scrambled sequences of 8 nt with approximately 50% GC content. Plasmid pRL-SV40 was a gift of Dr. David J. Shapiro.

### Effector plasmids

The effector plasmid pCMV-TTP (WT)-PUM-HD was GA-cloned from the following fragments: 2.2 kb and 2.4 kb pCMV5 fragments, GS-PUM-HD amplified from pTYB3-PUM1-HD, and TTP-GS amplified from cDNA (Open Biosystems catalog number MHS4768-99609440 [GenBank: BC009693.1]). pCMV-TTP (WT) and pCMV-PUM-HD have been assembled from the same vector backbone fragments, as well as TTP-stop or Flag-PUM-HD fragments, respectively (for primers, see Additional file 1: Table S4).

### Golden gate assembly of mutant effector plasmids

His-tagged PUF or 3xFlag-tagged TPUF constructs for *E. coli* or mammalian expression were assembled in pET28-GG-PUF or pCMV-TTP (C147R)-GG-PUF, respectively. Receiving vector of choice (50 ng) and 8 modules of choice (75 ng each) were combined with 1 μl T4 DNA ligase and 1 μl BsaI-HF in 10 μl 1× T4 DNA ligase buffer. The reactions were cycled 10 times for 5 min at 37°C and 10 min at 16°C, and a final incubation of 15 min at 37°C. TOP10 *E. coli* cells (Invitrogen) were then transformed with the cloning reactions and plated on LB plates (Cell Media Facility, UIUC) with either *kan* or *amp* selection, and supplemented with 10 μl 0.4 M IPTG (GoldBio) and 40 μl 20 mg/ml Bluo-Gal (Invitrogen) for blue-white screening. All the plasmids for *E. coli* expression were purified using Qiagen Qiaprep Spin Miniprep kit, and plasmids for mammalian expression were purified using Qiagen Plasmid Mini kit.

### Protein expression and purification

His-tagged recombinant PUF proteins were expressed in *E. coli* strain BL21 (DE3) (Novagen). The transformed BL21 cultures were grown in 100 ml LB until they reached an OD_600_ of 0.8, induced with 0.4 mM IPTG and expressed at 18°C, 250 RPM overnight. Bacterial pellets were resuspended in lysis buffer (25 mM Tris-HCl pH 7.5, 0.3 M NaCl, 0.5% Triton (Bio-Rad), 5% glycerol (Sigma), 1 mg/ml lysozyme (Sigma), and 0.002 U/μl DNase I) and lysed by sonication. The proteins were purified using Talon Spin Columns (Clontech), according to manufacturer’s instructions. The eluted protein was flash-frozen in 25% glycerol in dry ice and stored in aliquots at -80°C.

### Fluorescence polarization assay

RNA oligomers were modified with 6-carboxyfluorescein (IDT) at the 5′-end. To determine active protein fractions, we performed saturation assays for PUF proteins against their cognate RNA (a representative saturation curve is shown in Additional file 1: Figure S2a). High concentrations (100 nM) of RNA oligomers in fluorescence anisotropy buffer (20 mM Tris-HCl pH 7.5, 0.5 mM EDTA, 50 mM KCl, 0.1 mg/ml BSA) were mixed with various protein concentrations (determined by Bradford assay), and 200 μl protein-RNA mixtures were assayed (for fluorescence polarization measurements, see below) in black 96-well plates (Corning). The stoichiometric points were used to estimate the active protein fractions, which were determined to be 31% for PUF (WT), 30% for PUF (S2), 30% for PUF (S4), 33% for PUF (S6), and 29% for PUF (S6). Corrected active protein concentrations were used in the subsequent binding curves for the determination of the dissociation constants K_D_, where RNA oligomers (250 pM RNA for PUF (WT) and 1 nM RNA for PUF (S2)-PUF (S8)) in the fluorescence anisotropy buffer were mixed with various protein concentrations, and duplicates of 200 μl protein-RNA mixtures were assayed.

Fluorescence polarization measurements were taken on Tecan Infinite 200Pro using excitation and emission wavelengths of 485 nm and 535 nm, respectively. The fluorescence polarization values were converted to fluorescence anisotropy values using Equation 1, where A is anisotropy and P is polarization. The K_D_ was calculated by curve fitting on Origin 8.5 using Equation 2, where A is observed anisotropy value, A_f_ is anisotropy of free RNA, A_b_ is anisotropy of bound RNA, L_T_ is total ligand (RNA) concentration, and R_T_ is total receptor (protein) concentration.

(1) $$A=\frac{2P}{3-P}$$

(2) $$\begin{array}{ll} A= & A_{f}+\left(A_{b}-A_{f}\right)\\ & *\frac{\left(L_{T}+K_{D}+R_{T}\right)-{\sqrt{{\left(L_{T}+K_{D}+R_{T}\right)}^{2}-4L_{T}R}}_{T}}{2L_{T}} \end{array}$$

### Cell line transfection and dual luciferase assay

Transfection of HeLa cells (ATCC) was performed in triplicates in a 24-well plate format with Fugene-HD transfection reagent (Promega). Transfection mixtures contained 150 ng FL, 2 ng pRL-SV40, and 75 ng TPUF or equimolar amounts of other effector DNA constructs, and empty vector pCMV5 to 500 ng total. Cells were lysed in Passive Lysis Buffer (Promega) 48 h after transfection and FL and RL activities were measured in white 96-well plates (Greiner Bio One) using Dual-Glo Luciferase Assay System (Promega) with measurements taken on Analyst HT microplate reader at the High-Throughput Screening Facility at UIUC.

### RT-PCR

Total RNA was isolated from HeLa cells 48 hours after transfection using the RNeasy Mini Kit (Qiagen) following manufacturer’s instructions, and DNA was removed from samples with Turbo DNase (Life Technologies). RNA was reverse transcribed into cDNA with ProtoScript First Strand cDNA Synthesis kit (NEB) using the d(T)_23_VN primer. Reverse transcriptase was omitted in control samples. RT-PCR was performed using Power SYBR Green Master Mix (Life Technologies) with the 7900HT Fast Real-Time PCR System (Applied Biosystems). Reactions were carried out in triplicates in 20 μl reactions with 500 nM of each primer. The primer sequences for FL were 5′-GCGCGGAGGAGTTGTGTTTG and 5′-ATCTTTCCGCCCTTCTTGGC; and for RL 5′-GCAGCATATCTTG AACCATTC and 5′-TTGTACAACGTCAGGTTTACC. ΔΔCT method was used for RNA level analysis, where FL mRNA levels were normalized to RL mRNA, and FL_PBS (WT)_ mRNA levels were normalized to FL_Random_.

### VEGF induction and ELISA assay

For hypoxia-induced VEGFA, HEK293 cells were transfected in a 24-well plate format in triplicates with Fugene-HD. Transfection mixtures contained 500 ng TPUF DNA constructs or 350 ng pmaxGFP (Lonza) and 150 ng pCMV5. The cells were induced 24 h after transfection with 500 μM CoCl_2_, and the supernatant was collected for assay 24 hours after induction. For gene-switch-induced VEGFA, HEK293 cell line with retrovirally integrated DHB-inducible V24P-GS60 transcription activator was used [40]. The cells were transfected in a 24-well plate format in triplicates as above. The cells were induced 24 h after transfection with 100 nM DHB, in the presence of *pen*/*strep* (Gibco). The supernatant was collected 24 h after induction and subjected to ELISA. The assay was performed by pre-coating the 96-well clear plate with a goat anti-mouse antibody (Thermo Scientific) at 4°C overnight, and then following the instructions of human VEGF DuoSet kit (R & D Systems). The absorption readings were taken on a SpectraMax 340PC microplate reader. The cell monolayer was saved for Bradford assay.

### Bradford assay

The cell monolayers were lysed using RIPA lysis buffer. The protein concentrations were measured in technical duplicates by mixing 4 μl of cell lysate with 295 μl of Coomassie Plus Protein Assay Reagent (Thermo Scientific) in a 96-well clear plate. Quick Start Bovine Serum Albumine Standard Set (Bio-Rad) was used to build a protein standard curve. A_595_ was measured 5 min later using a SpectraMax 340PC microplate reader. The total protein concentrations measured by Bradford assay were used to normalize the VEGF concentrations.

### Western blotting

V24P-GS60-integrated 293 cells as well as HeLa cells were transfected in a 6-well plate format with Fugene-HD and 3 μg of effector plasmid. Cells were lysed using RIPA lysis buffer (Santa Cruz Biotech). The proteins were detected using mouse anti-Flag and anti-α-tubulin antibodies (GeneScript) and imaged using SuperSignal West Dura chemiluminescent substrate (Thermo Scientific).

## Abbreviations

PUF: Pumilio/fem-3 mRNA binding factor; RBP: RNA-binding protein; PUM-HD: Pumilio homology domain; PUM1: Pumilio 1; GG: Golden Gate; TTP: tristetraprolin; ARE: AU-rich element; PBS: PUF-binding site; TPUF: TTP (C147R)-PUM-HD; FL: firefly luciferase; RL: *Renilla* luciferase; VEGFA: vascular endothelial growth factor-A; DHB: 4,4′-dihydroxybenzyl; GA: Gibson Assembly.

## Competing interests

The authors declare that they have no competing interests.

## Authors’ contributions

ZA conceived and designed the study, carried out the experiments, analyzed and interpreted the data, and wrote the manuscript. CAD and HZ contributed to the experimental design, analysis and interpretation of the data. All authors have read and approved the final manuscript.

## Supplementary Material

Additional file 1: Figure S1 Confirmation through restriction enzyme digestion and gel-electrophoresis of GG assembled plasmids from randomly picked clones. First and last lanes, 1 kb DNA ladder (NEB). (a) KpnI and HindIII digestion of PUF (WT) clones assembled into pET28-GG-PUF receiving vector. 1 kb fragment contains the full length of the assembled PUF domain. (b) SalI and KpnI digestion of PUF (WT) clones assembled into pCMV-TTP-GG-PUF receiving vector. 1 kb fragment contains the assembled PUF domain region. **Figure S2**. Representative fluorescence anisotropy data for RNA binding to various PUF proteins. (a) Representative saturation curve of PUF (S4). (b) Binding curves of PUF (WT) (c) Binding curves of PUF (S2) (d) Binding curves of PUF (S4) (e) Binding curves of PUF (S6) (f) Binding curves of PUF (S8). Black, binding to cognate RNA. Red, binding to noncognate RNA. Each data point is represented by the mean ± SD. K_D_ values were calculated from nonlinear curve fitting. **Figure S3**. Schematics of the luciferase reporter assay and TPUF platform. (a) Schematic of full-length PUM1, TTP (WT), and TPUF constructs. CCCH, zinc finger domain; GS_L_ , glycine-serine linker. (b) Schematic of luciferase reporters. Orange boxes, PUF-binding sites. **Figure S4**. Dual luciferase assay showing TPUF (WT) repression of FL with increasing number of PBSs in the 3′ UTR of the reporter gene. Data represented as mean fold change relative to cells transfected with FL with no PBS ± SD: **P ≤ 0.01 (n=3, t test). **Figure S5**. Relative levels of FL/RL mRNA, normalized to FL_Ran_/RL mRNA in the presence of effectors. Fluorescence RT-PCR data were analyzed by ΔΔC_T_ method. Data represented as mean fold change relative to cells transfected with FL _Random_ (dashed line, unrepressed level) ± SD: n.s., not significant (n=3, t test). **Figure S6**. Dual luciferase assay showing FL reporter repression activity of TPUF (S4). Data represented as mean fold change relative to cells transfected with FL _Random_ ± SD: ***P ≤ 0.001 (n=3, t test). **Table S1**. GG library sequences (a) Aa sequences of WT and mutant modules. Black, WT aa. Red, mutant aa. (b) DNA sequences of WT and mutant modules. Black, WT sequence. Red, mutant nucleotides. **Table S2**. Primer list for GG library creation. **Table S3**. Primer list for FL cloning. **Table S4**. Primer list for effector plasmid cloning. Click here for file
