# Supplementary material for: Modular assembly of designer PUF proteins for specific post-transcriptional regulation of endogenous RNA
Source: J Biol Eng. 2014 Mar 1;8:7. doi: 10.1186/1754-1611-8-7 (PMC3943411; doi:10.1186/1754-1611-8-7)
Supplement: Additional file 1: Figure S1 — Confirmation through restriction enzyme digestion and gel-electrophoresis of GG assembled plasmids from randomly picked clones. First and last lanes, 1 kb DNA ladder (NEB). (a) KpnI and HindIII digestion of PUF (WT) clones assembled into pET28-GG-PUF receiving vector. 1 kb fragment contains the full length of the assembled PUF domain. (b) SalI and KpnI digestion of PUF (WT) clones assembled into pCMV-TTP-GG-PUF receiving vector. 1 kb fragment contains the assembled PUF domain region. Figure S2. Representative fluorescence anisotropy data for RNA binding to various PUF proteins. (a) Representative saturation curve of PUF (S4). (b) Binding curves of PUF (WT) (c) Binding curves of PUF (S2) (d) Binding curves of PUF (S4) (e) Binding curves of PUF (S6) (f) Binding curves of PUF (S8). Black, binding to cognate RNA. Red, binding to noncognate RNA. Each data point is represented by the mean ± SD. KD values were calculated from nonlinear curve fitting. Figure S3. Schematics of the luciferase reporter assay and TPUF platform. (a) Schematic of full-length PUM1, TTP (WT), and TPUF constructs. CCCH, zinc finger domain; GSL , glycine-serine linker. (b) Schematic of luciferase reporters. Orange boxes, PUF-binding sites. Figure S4. Dual luciferase assay showing TPUF (WT) repression of FL with increasing number of PBSs in the 3′ UTR of the reporter gene. Data represented as mean fold change relative to cells transfected with FL with no PBS ± SD: **P ≤ 0.01 (n=3, t test). Figure S5. Relative levels of FL/RL mRNA, normalized to FLRan/RL mRNA in the presence of effectors. Fluorescence RT-PCR data were analyzed by ΔΔCT method. Data represented as mean fold change relative to cells transfected with FL Random (dashed line, unrepressed level) ± SD: n.s., not significant (n=3, t test). Figure S6. Dual luciferase assay showing FL reporter repression activity of TPUF (S4). Data represented as mean fold change relative to cells transfected with FL Random ± SD: ***P ≤ 0.001 (n=3, t test). Table [file 1754-1611-8-7-S1.docx]

# Supplementary Information

**Modular assembly of designer PUF proteins for specific post-transcriptional regulation of endogenous RNA**

Zhanar Abil, Carl A. Denard, Huimin Zhao

# Supplementary Figures

Fig. S1. Confirmation through restriction enzyme digestion and gel-electrophoresis of GG assembled plasmids from randomly picked clones.

Fig. S2. Representative fluorescence anisotropy data for RNA binding to various PUF proteins.

Fig. S3. Schematics of the luciferase reporter assay and TPUF platform.

Fig. S4. Dual luciferase assay showing TPUF(WT) repression of FL with increasing number of PBSs in the 3′UTR of the reporter gene.

Fig. S5. Relative levels of FL/RL mRNA, normalized to FL_Ran_/RL mRNA in the presence of effectors.

Fig. S6. Dual luciferase assay showing FL reporter repression activity of TPUF(S4).

**Supplementary Tables**

Table S1. GG library sequences.

Table S2. Primer list for GG library creation.

Table S3. Primer list for FL cloning.

Table S4. Primer list for effector plasmid cloning.

# Supplementary Figures

Fig. S1. Confirmation through restriction enzyme digestion and gel-electrophoresis of GG assembled plasmids from randomly picked clones. First and last lanes, 1 kb DNA ladder (NEB). (a) KpnI and HindIII digestion of PUF(WT) clones assembled into pET28-GG-PUF receiving vector. 1 kb fragment contains the full length of the assembled PUF domain. (b) SalI and KpnI digestion of PUF(WT) clones assembled into pCMV-TTP-GG-PUF receiving vector. 1 kb fragment contains the assembled PUF domain region.

1kb

1kb

Fig. S2. Representative fluorescence anisotropy data for RNA binding to various PUF proteins. (a) Representative saturation curve of PUF(S4). (b) Binding curves of PUF(WT) (c) Binding curves of PUF(S2) (d) Binding curves of PUF(S4) (e) Binding curves of PUF(S6) (f) Binding curves of PUF(S8). Black, binding to cognate RNA. Red, binding to non-cognate RNA. Each data point is represented by the mean ± SD. K_D_ values were calculated from nonlinear curve fitting.

y=214847x+0.01796

R^2^=0.975

R^2^=0.994

R^2^=0.995

R^2^=0.996

R^2^=0.995

y=1914x+0.08881

Fig. S3. Schematics of the luciferase reporter assay and TPUF platform. (a) Schematic of full-length PUM1, TTP(WT), and TPUF constructs. CCCH, zinc finger domain; GS_L_ , glycine-serine linker. (PBS). (b) Schematic of luciferase reporters. Orange boxes, PUF binding sites

Fig. S4. Dual luciferase assay showing TPUF(WT) repression of FL with increasing number of PBSs in the 3′UTR of the reporter gene. Data represented as mean fold change relative to cells transfected with FL with no PBS ± SD: **P ≤ 0.01 (n=3, t test).

Fig. S5. Relative levels of FL/RL mRNA, normalized to FL_Ran_/RL mRNA in the presence of effectors. Fluorescence RT-PCR data were analyzed by ΔΔC_T_ method. Data represented as mean fold change relative to cells transfected with FL _Random_ (dashed line, unrepressed level) ± SD: n.s., not significant (n=3, t test).

Fig.S6. Dual luciferase assay showing FL reporter repression activity of TPUF(S4). Data represented as mean fold change relative to cells transfected with FL _Random_ ± SD: ***P ≤ 0.001 (n=3, t test)

Table S1. GG library sequences (a) Aa sequences of WT and mutant modules. Black, WT aa. Red, mutant aa. (b) DNA sequences of WT and mutant modules. Black, WT sequence. Red, mutant nucleotides.

a

| Repeat | Recognition | AA sequence |
| --- | --- | --- |
| 1′ |  | MGRSRLLEDFRNNRYPNLQLREIAG |
| 1 | A | HIMEFSQDQHGSRFIQLKLERATPAERQLVFNEILQ |
| 1 | G | HIMEFSQDQHGSRFIELKLERATPAERQLVFNEILQ |
| 1 | U | HIMEFSQDQHGNRFIQLKLERATPAERQLVFNEILQ |
| 1 | C | HIMEFSQDQHGSRFIRLKLERATPAERQLVFNEILQ |
| 2 | A | AAYQLMVDVFGCYVIQKFFEFGSLEQKLALAERIRG |
| 2 | G | AAYQLMVDVFGSYVIEKFFEFGSLEQKLALAERIRG |
| 2 | U | AAYQLMVDVFGNYVIQKFFEFGSLEQKLALAERIRG |
| 2 | C | AAYQLMVDVFGSYVIRKFFEFGSLEQKLALAERIRG |
| 3 | A | HVLSLALQMYGCRVIQKALEFIPSDQQNEMVRELDG |
| 3 | G | HVLSLALQMYGSRVIEKALEFIPSDQQNEMVRELDG |
| 3 | U | HVLSLALQMYGNRVIQKALEFIPSDQQNEMVRELDG |
| 3 | C | HVLSLALQMYGSRVIRKALEFIPSDQQNEMVRELDG |
| 4 | A | HVLKCVKDQNGCHVVQKCIECVQPQSLQFIIDAFKG |
| 4 | G | HVLKCVKDQNGSHVVEKCIECVQPQSLQFIIDAFKG |
| 4 | U | HVLKCVKDQNGNHVVQKCIECVQPQSLQFIIDAFKG |
| 4 | C | HVLKCVKDQNGSHVVRKCIECVQPQSLQFIIDAFKG |
| 4 | A | HVLKCVKDQNGCYVVQKCIECVQPQSLQFIIDAFKG |
| 4 | G | HVLKCVKDQNGSYVVEKCIECVQPQSLQFIIDAFKG |
| 4 | U | HVLKCVKDQNGNYVVQKCIECVQPQSLQFIIDAFKG |
| 4 | C | HVLKCVKDQNGSYVVRKCIECVQPQSLQFIIDAFKG |
| 5 | A | QVFALSTHPYGCRVIQRILEHCLPDQTLPILEELHQ |
| 5 | G | QVFALSTHPYGSRVIERILEHCLPDQTLPILEELHQ |
| 5 | U | QVFALSTHPYGNRVIQRILEHCLPDQTLPILEELHQ |
| 5 | C | QVFALSTHPYGSRVIRRILEHCLPDQTLPILEELHQ |
| 6 | A | HTEQLVQDQYGCYVIQHVLEHGRPEDKSKIVAEIRG |
| 6 | G | HTEQLVQDQYGSYVIEHVLEHGRPEDKSKIVAEIRG |
| 6 | U | HTEQLVQDQYGNYVIQHVLEHGRPEDKSKIVAEIRG |
| 6 | C | HTEQLVQDQYGSYVIRHVLEHGRPEDKSKIVAEIRG |
| 7 | A | NVLVLSQHKFACNVVQKCVTHASRTERAVLIDEVCTMNDGPHS |
| 7 | G | NVLVLSQHKFASNVVEKCVTHASRTERAVLIDEVCTMNDGPHS |
| 7 | U | NVLVLSQHKFANNVVQKCVTHASRTERAVLIDEVCTMNDGPHS |
| 7 | C | NVLVLSQHKFASYVVRKCVTHASRTERAVLIDEVCTMNDGPHS |
| 8 | A | ALYTMMKDQYACYVVQKMIDVAEPGQRKIVMHKIRP |
| 8 | G | ALYTMMKDQYASYVVEKMIDVAEPGQRKIVMHKIRP |
| 8 | U | ALYTMMKDQYANYVVQKMIDVAEPGQRKIVMHKIRP |
| 8 | C | ALYTMMKDQYASYVVRKMIDVAEPGQRKIVMHKIRP |
| 8′ |  | HIATLRKYTYGKHILAKLEKYYMKNGVDLG |

b

| Re-peat | | Recog-nition | | Over-hang | DNA sequence | Over-hang |
| --- | --- | --- | --- | --- | --- | --- |
| 1′ |  | |  | | ATGGGCCGCAGCCGCCTTTTGGAAGATTTTCGAAACAACCGGTACCCCAATTTACAACTGCGGGAGATTGCCGGA |  |
| 1 | | A | | CGGA | CATATAATGGAATTTTCCCAAGACCAGCATGGGTCCAGATTCATTCAGCTGAAACTGGAGCGTGCCACACCAGCTGAGCGCCAGCTTGTCTTCAATGAAATCCTCCAG |  |
| 1 | | G | | CGGA | CATATAATGGAATTTTCCCAAGACCAGCATGGGTCCAGATTCATTGAGCTGAAACTGGAGCGTGCCACACCAGCTGAGCGCCAGCTTGTCTTCAATGAAATCCTCCAG |  |
| 1 | | U | | CGGA | CATATAATGGAATTTTCCCAAGACCAGCATGGGAACAGATTCATTCAGCTGAAACTGGAGCGTGCCACACCAGCTGAGCGCCAGCTTGTCTTCAATGAAATCCTCCAG |  |
| 1 | | C | | CGGA | CATATAATGGAATTTTCCCAAGACCAGCATGGGTCCAGATTCATTCGCCTGAAACTGGAGCGTGCCACACCAGCTGAGCGCCAGCTTGTCTTCAATGAAATCCTCCAG |  |
| 2 | | A | | CCAG | GCTGCCTACCAACTCATGGTGGATGTGTTTGGTTGTTACGTCATTCAGAAGTTCTTTGAATTTGGCAGTCTTGAACAGAAGCTGGCTTTGGCAGAACGGATTCGAGGT |  |
| 2 | | G | | CCAG | GCTGCCTACCAACTCATGGTGGATGTGTTTGGTAGTTACGTCATTGAGAAGTTCTTTGAATTTGGCAGTCTTGAACAGAAGCTGGCTTTGGCAGAACGGATTCGAGGT |  |
| 2 | | U | | CCAG | GCTGCCTACCAACTCATGGTGGATGTGTTTGGTAATTACGTCATTCAGAAGTTCTTTGAATTTGGCAGTCTTGAACAGAAGCTGGCTTTGGCAGAACGGATTCGAGGT |  |
| 2 | | C | | CCAG | GCTGCCTACCAACTCATGGTGGATGTGTTTGGTAGTTACGTCATTCGCAAGTTCTTTGAATTTGGCAGTCTTGAACAGAAGCTGGCTTTGGCAGAACGGATTCGAGGT |  |
| 3 | | A | | AGGT | CACGTCCTGTCATTGGCACTACAGATGTATGGCTGCCGTGTTATCCAGAAAGCTCTTGAGTTTATTCCTTCAGACCAGCAGAATGAGATGGTTCGGGAACTAGATGGC |  |
| 3 | | G | | AGGT | CACGTCCTGTCATTGGCACTACAGATGTATGGCTCCCGTGTTATCGAGAAAGCTCTTGAGTTTATTCCTTCAGACCAGCAGAATGAGATGGTTCGGGAACTAGATGGC |  |
| 3 | | U | | AGGT | CACGTCCTGTCATTGGCACTACAGATGTATGGCAACCGTGTTATCCAGAAAGCTCTTGAGTTTATTCCTTCAGACCAGCAGAATGAGATGGTTCGGGAACTAGATGGC |  |
| 3 | | C | | AGGT | CACGTCCTGTCATTGGCACTACAGATGTATGGCTCCCGTGTTATCCGCAAAGCTCTTGAGTTTATTCCTTCAGACCAGCAGAATGAGATGGTTCGGGAACTAGATGGC |  |
| 4 | | A | | TGGC | CATGTCTTGAAGTGTGTGAAAGATCAGAATGGCTGTCACGTGGTTCAGAAATGCATTGAATGTGTACAGCCCCAGTCTTTGCAATTTATCATCGATGCGTTTAAGGGC | CAGG |
| 4 | | G | | TGGC | CATGTCTTGAAGTGTGTGAAAGATCAGAATGGCAGTCACGTGGTTGAGAAATGCATTGAATGTGTACAGCCCCAGTCTTTGCAATTTATCATCGATGCGTTTAAGGGA | CAGG |
| 4 | | U | | TGGC | CATGTCTTGAAGTGTGTGAAAGATCAGAATGGCAATCACGTGGTTCAGAAATGCATTGAATGTGTACAGCCCCAGTCTTTGCAATTTATCATCGATGCGTTTAAGGGA | CAGG |
| 4 | | C | | TGGC | CATGTCTTGAAGTGTGTGAAAGATCAGAATGGCAGTCACGTGGTTCGCAAATGCATTGAATGTGTACAGCCCCAGTCTTTGCAATTTATCATCGATGCGTTTAAGGGA | CAGG |
| 4 | | A | | TGGC | CATGTCTTGAAGTGTGTGAAAGATCAGAATGGCTGTTACGTGGTTCAGAAATGCATTGAATGTGTACAGCCCCAGTCTTTGCAATTTATCATCGATGCGTTTAAGGGA | CAGG |
| 4 | | G | | TGGC | CATGTCTTGAAGTGTGTGAAAGATCAGAATGGCAGTTACGTGGTTGAGAAATGCATTGAATGTGTACAGCCCCAGTCTTTGCAATTTATCATCGATGCGTTTAAGGGA | CAGG |
| 4 | | U | | TGGC | CATGTCTTGAAGTGTGTGAAAGATCAGAATGGCAATTACGTGGTTCAGAAATGCATTGAATGTGTACAGCCCCAGTCTTTGCAATTTATCATCGATGCGTTTAAGGGA | CAGG |
| 4 | | C | | TGGC | CATGTCTTGAAGTGTGTGAAAGATCAGAATGGCAGTTACGTGGTTCGCAAATGCATTGAATGTGTACAGCCCCAGTCTTTGCAATTTATCATCGATGCGTTTAAGGGA | CAGG |
| 5 | | A | |  | CAGGTATTTGCCTTATCCACACATCCTTATGGCTGCCGAGTGATTCAGAGAATCCTGGAGCACTGTCTCCCTGACCAGACACTCCCTATTTTAGAGGAGCTTCACCAG | CACA |
| 5 | | G | |  | CAGGTATTTGCCTTATCCACACATCCTTATGGCTCCCGAGTGATTGAGAGAATCCTGGAGCACTGTCTCCCTGACCAGACACTCCCTATTTTAGAGGAGCTTCACCAG | CACA |
| 5 | | U | |  | CAGGTATTTGCCTTATCCACACATCCTTATGGCAACCGAGTGATTCAGAGAATCCTGGAGCACTGTCTCCCTGACCAGACACTCCCTATTTTAGAGGAGCTTCACCAG | CACA |
| 5 | | C | |  | CAGGTATTTGCCTTATCCACACATCCTTATGGCTCCCGAGTGATTCGCAGAATCCTGGAGCACTGTCTCCCTGACCAGACACTCCCTATTTTAGAGGAGCTTCACCAG | CACA |
| 6 | | A | |  | CACACAGAGCAGCTTGTACAGGATCAATATGGATGTTATGTAATCCAACATGTACTGGAGCACGGTCGTCCTGAGGATAAAAGCAAAATTGTAGCAGAAATCCGAGGC | AATG |
| 6 | | G | |  | CACACAGAGCAGCTTGTACAGGATCAATATGGAAGTTATGTAATCGAACATGTACTGGAGCACGGTCGTCCTGAGGATAAAAGCAAAATTGTAGCAGAAATCCGAGGC | AATG |
| 6 | | U | |  | CACACAGAGCAGCTTGTACAGGATCAATATGGAAATTATGTAATCCAACATGTACTGGAGCACGGTCGTCCTGAGGATAAAAGCAAAATTGTAGCAGAAATCCGAGGC | AATG |
| 6 | | C | |  | CACACAGAGCAGCTTGTACAGGATCAATATGGAAGTTATGTAATCCGCCATGTACTGGAGCACGGTCGTCCTGAGGATAAAAGCAAAATTGTAGCAGAAATCCGAGGC | AATG |
| 7 | | A | |  | AATGTACTTGTATTGAGTCAGCACAAATTTGCATGCAATGTTGTGCAGAAGTGTGTTACTCACGCCTCACGTACGGAGCGCGCTGTGCTCATCGATGAGGTGTGCACCATGAACGACGGTCCCCACAGT | GCCT |
| 7 | | G | |  | AATGTACTTGTATTGAGTCAGCACAAATTTGCAAGCAATGTTGTGGAGAAGTGTGTTACTCACGCCTCACGTACGGAGCGCGCTGTGCTCATCGATGAGGTGTGCACCATGAACGACGGTCCCCACAGT | GCCT |
| 7 | | U | |  | AATGTACTTGTATTGAGTCAGCACAAATTTGCAAACAATGTTGTGCAGAAGTGTGTTACTCACGCCTCACGTACGGAGCGCGCTGTGCTCATCGATGAGGTGTGCACCATGAACGACGGTCCCCACAGT | GCCT |
| 7 | | C | |  | AATGTACTTGTATTGAGTCAGCACAAATTTGCAAGCTATGTTGTGCGCAAGTGTGTTACTCACGCCTCACGTACGGAGCGCGCTGTGCTCATCGATGAGGTGTGCACCATGAACGACGGTCCCCACAGT | GCCT |
| 8 | | A | |  | GCCTTATACACCATGATGAAGGACCAGTATGCCTGCTACGTGGTCCAGAAGATGATTGACGTGGCGGAGCCAGGCCAGCGGAAGATCGTCATGCATAAGATCCGACCC |  |
| 8 | | G | |  | GCCTTATACACCATGATGAAGGACCAGTATGCCAGCTACGTGGTCGAGAAGATGATTGACGTGGCGGAGCCAGGCCAGCGGAAGATCGTCATGCATAAGATCCGACCC |  |
| 8 | | U | |  | GCCTTATACACCATGATGAAGGACCAGTATGCCAACTACGTGGTCCAGAAGATGATTGACGTGGCGGAGCCAGGCCAGCGGAAGATCGTCATGCATAAGATCCGACCC |  |
| 8 | | C | |  | GCCTTATACACCATGATGAAGGACCAGTATGCCAGCTACGTGGTCCGCAAGATGATTGACGTGGCGGAGCCAGGCCAGCGGAAGATCGTCATGCATAAGATCCGACCC |  |
| 8′ | |  | | ACCC | CACATCGCAACTCTTCGTAAGTACACCTATGGCAAGCACATTCTGGCCAAGCTGGAGAAGTACTACATGAAGAACGGTGTTGACTTAGGG |  |

Table S2. Primer list for GG library creation.

| **Primer name** | **Primer sequence** | **Amplicon** |
| --- | --- | --- |
|  | **Gibson Assembly of the intermediate plasmid** |  |
| pUC19-CAT-F | GGGGTCTGACGCTCAGTGGAACGAA CTTTCGAATTTCTGCCATTCATCCGC | CAT |
| CAT-pUC19-R | TCTCCTTACGCATCTGTGCGGTATT TGTGACGGAAGATCACTTCGCAG | CAT |
| CAT-pUC19-F | TTCTGCGAAGTGATCTTCCGTCACA AATACCGCACAGATGCGTAAGGAG | pNEB193 |
| pUC19-CAT-R | ATAAGCGGATGAATGGCAGAAATTCGAAAG TTCGTTCCACTGAGCGTCAGAC | pNEB193 |
|  |  |  |
|  | ***Sac*I-*Hind*III cloning of WT Golden Gate modules** |  |
| 1PUM-F | AAGTGAGCTCGGTCTCA C GGACATATAATGGAATTTTCCCAAGACCAGC | R1 (1SQ) |
| 2PUM-F | AAGTGAGCTCGGTCTCA CCAGGCTGCCTACCAACTCATG | R2 (2NQ) |
| 3PUM-F | AAGTGAGCTCGGTCTCA AGGT CACGTCCTGTCATTGGCACTAC | R3 (3CQ) |
| 4PUM-F | AAGTGAGCTCGGTCTCA TGGCCATGTCTTGAAGTGTGTGAAAG | R4 (4NQ) |
| 5PUM-F | AAGTGAGCTCGGTCTCA CAGGTATTTGCCTTATCCACACATCCTTATG | R5 (5CQ) |
| 6PUM-F | AAGTGAGCTCGGTCTCA CACACAGAGCAGCTTGTACAGG | R6 (6NQ) |
| 7PUM-F | AAGTGAGCTCGGTCTCA AATGTACTTGTATTGAGTCAGCACAAATTTGC | R7 (7SE) |
| 8-PUM-F | AAGTGAGCTCGGTCTCA GCCTTATACACCATGATGAAGGACCAG | R8 (8NQ) |
| 1PUM-R | TTCTAAGCTTGGTCTCT CTGGAGGATTTCATTGAAGACAAGCTGG | R1 (1SQ) |
| 2PUM-R | TTCTAAGCTTGGTCTCT ACCTCGAATCCGTTCTGCCAAAGC | R2 (2NQ) |
| 3PUM-R | TTCTAAGCTTGGTCTCT GCCATCTAGTTCCCGAACCATCTC | R3 (3CQ) |
| 4PUM-R | TTCTAAGCTTGGTCTCT CCTGTCCCTTAAACGCATCGATGATAAATTG | R4 (4NQ) |
| 5PUM-R | TTCTAAGCTTGGTCTCT TGTGCTGGTGAAGCTCCTCTAAAATAGG | R5 (5CQ) |
| 6PUM-R | TTCTAAGCTTGGTCTCT CATTGCCTCGGATTTCTGCTACAATTTTGC | R6 (6NQ) |
| 7PUM-R | TTCTAAGCTTGGTCTCT AGGCACTGTGGGGACCG | R7 (7SE) |
| 8PUM-R | TTCTAAGCTTGGTCTCT GGGT CGGATCTTATGCATGACGATCTTCCG | R8 (8NQ) |
|  |  |  |
|  | **Gibson Assembly of mutant Golden Gate modules** |  |
| 1Rev | CCCATGCTGGTCTTGGGAAAATTCC | All R1′s |
| 1SE-For | GGAATTTTCCCAAGACCAGCATGGGTCCAGATTCATTGAGCTGAAACTGGAGCGTGCCAC | 1SE |
| 1NQ-For | GGAATTTTCCCAAGACCAGCATGGGAACAGATTCATTCAGCTGAAACTGGAGCGTGCCAC | 1NQ |
| 1SR-For | GGAATTTTCCCAAGACCAGCATGGGTCCAGATTCATTCGCCTGAAACTGGAGCGTGCCAC | 1SR |
|  |  |  |
| 2Rev | ACCAAACACATCCACCATGAGTTGG | All R2′s |
| 2CQ-For | CCAACTCATGGTGGATGTGTTTGGTTGTTACGTCATTCAGAAGTTCTTTGAATTTGGCAG | 2CQ |
| 2SE-For | CCAACTCATGGTGGATGTGTTTGGTAGTTACGTCATTGAGAAGTTCTTTGAATTTGGCAG | 2SE |
| 2SR-For | CCAACTCATGGTGGATGTGTTTGGTAGTTACGTCATTCGCAAGTTCTTTGAATTTGGCAG | 2SR |
|  |  |  |
| 3Rev | GCCATACATCTGTAGTGCCAATGA | All R3′s |
| 3SE-For | TCATTGGCACTACAGATGTATGGCTCCCGTGTTATCGAGAAAGCTCTTGAGTTTATTCCT | 3SE |
| 3NQ-For | TCATTGGCACTACAGATGTATGGCAACCGTGTTATCCAGAAAGCTCTTGAGTTTATTCCT | 3NQ |
| 3SR-For | TCATTGGCACTACAGATGTATGGCTCCCGTGTTATCCGCAAAGCTCTTGAGTTTATTCCT | 3SR |
|  |  |  |
| 4Rev | GCCATTCTGATCTTTCACACACTTC | All R4′s |
| 4CQ-For | GAAGTGTGTGAAAGATCAGAATGGCTGTCACGTGGTTCAGAAATGCATTGAATGTGTACA | 4CQ |
| 4SE-For | GAAGTGTGTGAAAGATCAGAATGGCAGTCACGTGGTTGAGAAATGCATTGAATGTGTACA | 4SE |
| 4SR-For | GAAGTGTGTGAAAGATCAGAATGGCAGTCACGTGGTTCGCAAATGCATTGAATGTGTACA | 4SR |
| 4CYQ-For | GAAGTGTGTGAAAGATCAGAATGGCTGTTACGTGGTTCAGAAATGCATTGAATGTGTACA | 4CYQ |
| 4SYE-For | GAAGTGTGTGAAAGATCAGAATGGCAGTTACGTGGTTGAGAAATGCATTGAATGTGTACA | 4SYE |
| 4NYQ-For | GAAGTGTGTGAAAGATCAGAATGGCAATTACGTGGTTCAGAAATGCATTGAATGTGTACA | 4NYQ |
| 4SYR-For | GAAGTGTGTGAAAGATCAGAATGGCAGTTACGTGGTTCGCAAATGCATTGAATGTGTACA | 4SYR |
|  |  |  |
| 5Rev | GCCATAAGGATGTGTGGATAAGGC | All R5′s |
| 5SE-For | GCCTTATCCACACATCCTTATGGCTCCCGAGTGATTGAGAGAATCCTGGAGCACTGTCTC | 5SE |
| 5NQ-For | GCCTTATCCACACATCCTTATGGCAACCGAGTGATTCAGAGAATCCTGGAGCACTGTCTC | 5NQ |
| 5SR-For | GCCTTATCCACACATCCTTATGGCTCCCGAGTGATTCGCAGAATCCTGGAGCACTGTCTC | 5SR |
|  |  |  |
| 6Rev | TCCATATTGATCCTGTACAAGCTGCTC | All R6′s |
| 6CQ-For | GAGCAGCTTGTACAGGATCAATATGGATGTTATGTAATCCAACATGTACTGGAGCACGGT | 6CQ |
| 6SE-For | GAGCAGCTTGTACAGGATCAATATGGAAGTTATGTAATCGAACATGTACTGGAGCACGGT | 6SE |
| 6SR-For | GAGCAGCTTGTACAGGATCAATATGGAAGTTATGTAATCCGCCATGTACTGGAGCACGGT | 6SR |
|  |  |  |
| 7Rev | TGCAAATTTGTGCTGACTCAATACAA | All R7′s |
| 7CQ-For | TTGTATTGAGTCAGCACAAATTTGCATGCAATGTTGTGCAGAAGTGTGTTACTCACGCCT | 7CQ |
| 7NQ-For | TTGTATTGAGTCAGCACAAATTTGCAAACAATGTTGTGCAGAAGTGTGTTACTCACGCCT | 7NQ |
| 7SYR-For | TTGTATTGAGTCAGCACAAATTTGCAAGCTATGTTGTGCGCAAGTGTGTTACTCACGCCT | 7SYR |
|  |  |  |
| 8Rev | GGCATACTGGTCCTTCATCATGGT | All R8′s |
| 8CQ-For | ACCATGATGAAGGACCAGTATGCCTGCTACGTGGTCCAGAAGATGATTGACGTGGCGGAG | 8CQ |
| 8SE-For | ACCATGATGAAGGACCAGTATGCCAGCTACGTGGTCGAGAAGATGATTGACGTGGCGGAG | 8SE |
| 8SR-For | ACCATGATGAAGGACCAGTATGCCAGCTACGTGGTCCGCAAGATGATTGACGTGGCGGAG | 8SR |
|  |  |  |
|  | **Gibson Assembly of pET28-GG-PUF receiving vector** |  |
| pET28-1′-F | CTGGTGCCGCGCGGCAGCCA T GGCCGCAGCCGCCTTT | R1′ |
| 1′-LacZi-R | CATCTGTGCGG GGTCTCT TCCGGCAATCTCCCGCAGTTGTAAATTGG | R1′ |
| 1′-lacZ-F | GAGATTGCCGGA AGAGACC CCGCACAGATGCGTAAGGAG | LacZ |
| lacZ-8′i-R | TGCGATGTGGGGT TGAGACC GACTGGAAAGCGGGCAGTGAG | LacZ |
| lacZ-8′i-F | CGCTTTCCAGTC GGTCTCA A CCCCACATCGCAACTCTTCG | 8′ |
| 8′-pET28-R | CGAGTGCGGCCGCAAGCTTG TTA CCCTAAGTCAACACCGTTCTTCATGT | 8′ |
|  |  |  |
|  | **Gibson Assembly of pCMV-TTP-GG-PUF vector** |  |
| 1′-lacZ-F | GAGATTGCCGGA AGAGACC CCGCACAGATGCGTAAGGAG | LacZ |
| lacZ-8′i-R | TGCGATGTGGGGT TGAGACC GACTGGAAAGCGGGCAGTGAG | LacZ |
| lacZ-8′i-F | CGCTTTCCAGTC GGTCTCA A CCCCACATCGCAACTCTTCG | Fragment 1 |
| pGH PA -BsaI R | TAGGAGTTGGAGTTCAGCCTGGCCAATATGG | Fragment 1 |
| pGH PA -BsaI F | TTGGCCAGGCTG AA CTCCAACTCCTAATCTC | Fragment 2 |
| Amp -BsaI R | AGCCGGTGAGCGTGGATCTCGCGGTATC | Fragment 2 |
| Amp -BsaI F | ATGATACCGCGAGA T CCACGCTCACCG | Fragment 3 |
| TTP-BsaI R | CTGGGGTGGGATCTCTTCGAGCCA | Fragment 3 |
| TTP -BsaI F | CTCGAAGAGA T CCCACCCCAGTC | Fragment 4 |
| 1′-LacZi-R | CATCTGTGCGG GGTCTCT TCCGGCAATCTCCCGCAGTTGTAAATTGG | Fragment 4 |
|  |  |  |
|  | **Gibson Assembly of pCMV-TTP(C147R)-PUF receiving vector** |  |
| 3xFlag1F | GTGGGAGGTCTATATAAGCccaccATGGACTACAAAGACCATGACGGTGATTATAAAGAT | 3xFlag |
| 3xFlag2R | CATCGTCATCCTTGTAATCGATGTCATGATCTTTATAATCACCGTCATGGTCTTTG | 3xFlag |
| 3xFlag3F | CAAAGACCATGACGGTGATTATAAAGATCATGACATCGATTACAAGGATGACGATG | 3xFlag |
| 3xFlag4R | CTCTCGTAGATGGCAGTCAGATCCATCTTGTCATCGTCATCCTTGTAATCGATGTCATGA | 3xFlag |
| 3xFlag | Primer extension product from primers 3xFlag(1F-4R) above | 0.5kb fragment |
| TTP_C147R-R | AGGTAGAACTTGTGACGGAGTTCCGTCTTG | 0.5kb fragment |
| TTP_C147R-F | CAAGACGGAACTCCGTCACAAGTTCTACCT | 3.5 kb fragment |
| pCMV5-3235-R | CGCTGAGATAGGTGCCTCACTG | 3.5 kb fragment |
| pCMV5-3183-F | AACTTGGTCTGACAGTTACCAATGCTT | 2.4 kb fragment |
| pCMV896R | CAT GGTGG GCTTATATAGACCTCCCAC | 2.4 kb fragment |

Table S3. Primer list for FL cloning

| **Primer name** | **Primer sequence** | **Amplicon** |
| --- | --- | --- |
|  | ***Sac*I-*Kpn*I cloning of pCMV-Fluc** |  |
| SacI-Fluc-F | AGTC GAGCTC CCACCATGGAAGACGCCAAAAACATAAAG | Firefly luciferase |
| KpnI-Fluc-R | CTTAGGTACCCGACTCTAGAATTACACGGCGATCTTTC | Firefly luciferase |
|  |  |  |
|  | **Gibson Assembly of pCMV-Fluc-Random and pCMV-Fluc-10xPBS plasmids** |  |
| Fluc-random-1F | TATCGATAAGCTTGCATGCCTGCAGGGGATAGTAGACGTGAGACCGGAGCA | 3′UTR random |
| Fluc-random-2R | CCTATCGCTCGCTGGTGTGTAAGTGACATATGGGTTTGGGTGCTCCGGTCTCACGTCT | 3′UTR random |
| Fluc-random-3F | ACACCAGCGAGCGATAGGCACTGCCAAAGTGTATAAGGGGTCCGAGAGCTGGAAGGGA | 3′UTR random |
| Fluc-random-4R | TGCATGGAGGTTGGCCAGATCCTTCATAAGGAGATAAGGATCCCTTCCAGCTCTCGGA | 3′UTR random |
| Fluc-random-5F | CTGGCCAACCTCCATGCAGAGTGTCAAAGGAGGCCAGTGTGTGGCAGCCAGCATCTCG | 3′UTR random |
| Fluc-random-6R | GGGGTCACAGGGATGCCACCATCGGGTTCGAGATGCTGGCTGCCAC | 3′UTR random |
|  |  |  |
| Fluc-WT-1F | TATCGATAAGCTTGCATGCCTGCAGTGTATATAAGACGTGAGACCGGAGCA | 10xPBS(WT) |
| Fluc-WT-2R | CCTATCGCTCGCTGGTGTTATATACACATATGTATATACATGCTCCGGTCTCACGTCT | 10xPBS(WT) |
| Fluc-WT-3F | ACACCAGCGAGCGATAGGTGTATATAAAGTGTTGTATATATCCGAGAGCTGGAAGGGA | 10xPBS(WT) |
| Fluc-WT-4R | TGCATGGAGGTTGGCCAGTATATACATAAGGATATATACATCCCTTCCAGCTCTCGGA | 10xPBS(WT) |
| Fluc-WT-5F | CTGGCCAACCTCCATGCATGTATATAAAGGAGTGTATATAGTGGCAGCCAGCATCTCG | 10xPBS(WT) |
| Fluc-WT-6R | GGGGTCACAGGGATGCCACCTATATACACGAGATGCTGGCTGCCAC | 10xPBS(WT) |
|  |  |  |
| FlucARE_1F | TATCGATAAGCTTGCATGCCTGCAGTTATTTATTAGACGTGAGACCGGAGCA | 10xARE |
| FlucARE_2R | CCTATCGCTCGCTGGTGTAATAAATAACATATGAATAAATAATGCTCCGGTCTCACGTCT | 10xARE |
| FlucARE_3F | ACACCAGCGAGCGATAGGTTATTTATTAAGTGTTTATTTATTTCCGAGAGCTGGAAGGGA | 10xARE |
| FlucARE_4R | TGCATGGAGGTTGGCCAGAATAAATAATAAGGAAATAAATAATCCCTTCCAGCTCTCGGA | 10xARE |
| FlucARE_5F | CTGGCCAACCTCCATGCATTATTTATTAAGGAGTTATTTATTGTGGCAGCCAGCATCTCG | 10xARE |
| FlucARE_6R | GGGGTCACAGGGATGCCACCAATAAATAACGAGATGCTGGCTGCCAC | 10xARE |
|  |  |  |
| G-A8G-10x-1F | TATCGATAAGCTTGCATGCCTGCAGTGTATATGAGACGTGAGACCGGAGCA | 10xPBS(A8G) |
| G-A8G-10x-2R | CCTATCGCTCGCTGGTGTCATATACACATATGCATATACATGCTCCGGTCTCACGTCT | 10xPBS(A8G) |
| G-A8G-10x-3F | ACACCAGCGAGCGATAGGTGTATATGAAGTGTTGTATATGTCCGAGAGCTGGAAGGGA | 10xPBS(A8G) |
| G-A8G-10x-4R | TGCATGGAGGTTGGCCAGCATATACATAAGGACATATACATCCCTTCCAGCTCTCGGA | 10xPBS(A8G) |
| G-A8G-10x-5F | CTGGCCAACCTCCATGCATGTATATGAAGGAGTGTATATGGTGGCAGCCAGCATCTCG | 10xPBS(A8G) |
| G-A8G-10x-6R | GGGGTCACAGGGATGCCACCCATATACACGAGATGCTGGCTGCCAC | 10xPBS(A8G) |
|  |  |  |
| G-GU23UG-10x-1F | TATCGATAAGCTTGCATGCCTGCAGTTGATATAAGACGTGAGACCGGAGCA | 10xPBS(GU/UG) |
| G-GU23UG-10x-2R | CCTATCGCTCGCTGGTGTTATATCAACATATGTATATCAATGCTCCGGTCTCACGTCT | 10xPBS(GU/UG) |
| G-GU23UG-10x-3F | ACACCAGCGAGCGATAGGTTGATATAAAGTGTTTGATATATCCGAGAGCTGGAAGGGA | 10xPBS(GU/UG) |
| G-GU23UG-10x-4R | TGCATGGAGGTTGGCCAGTATATCAATAAGGATATATCAATCCCTTCCAGCTCTCGGA | 10xPBS(GU/UG) |
| G-GU23UG-10x-5F | CTGGCCAACCTCCATGCATTGATATAAAGGAGTTGATATAGTGGCAGCCAGCATCTCG | 10xPBS(GU/UG) |
| G-GU23UG-10x-6R | GGGGTCACAGGGATGCCACCTATATCAACGAGATGCTGGCTGCCAC | 10xPBS(GU/UG) |
|  |  |  |
| Fluc-S2_1F | TATCGATAAGCTTGCATGCCTGCAGAGTATATTAGACGTGAGACCGGAGCA | 10xPBS(S2) |
| Fluc-S2_2R | CCTATCGCTCGCTGGTGTAATATACTCATATGAATATACTTGCTCCGGTCTCACGTCT | 10xPBS(S2) |
| Fluc-S2_3F | ACACCAGCGAGCGATAGGAGTATATTAAGTGTAGTATATTTCCGAGAGCTGGAAGGGA | 10xPBS(S2) |
| Fluc-S2_4R | TGCATGGAGGTTGGCCAGAATATACTTAAGGAAATATACTTCCCTTCCAGCTCTCGGA | 10xPBS(S2) |
| Fluc-S2_5F | CTGGCCAACCTCCATGCAAGTATATTAAGGAGAGTATATTGTGGCAGCCAGCATCTCG | 10xPBS(S2) |
| Fluc-S2_6R | GGGGTCACAGGGATGCCACCAATATACTCGAGATGCTGGCTGCCAC | 10xPBS(S2) |
|  |  |  |
| Fluc-S4_1F | TATCGATAAGCTTGCATGCCTGCAGTGATATTAAGACGTGAGACCGGAGCA | 10xPBS(S4) |
| Fluc-S4_2R | CCTATCGCTCGCTGGTGTTAATATCACATATGTAATATCATGCTCCGGTCTCACGTCT | 10xPBS(S4) |
| Fluc-S4_3F | ACACCAGCGAGCGATAGGTGATATTAAAGTGTTGATATTATCCGAGAGCTGGAAGGGA | 10xPBS(S4) |
| Fluc-S4_4R | TGCATGGAGGTTGGCCAGTAATATCATAAGGATAATATCATCCCTTCCAGCTCTCGGA | 10xPBS(S4) |
| Fluc-S4_5F | CTGGCCAACCTCCATGCATGATATTAAAGGAGTGATATTAGTGGCAGCCAGCATCTCG | 10xPBS(S4) |
| Fluc-S4_6R | GGGGTCACAGGGATGCCACCTAATATCACGAGATGCTGGCTGCCAC | 10xPBS(S4) |
|  |  |  |
| Fluc-S6_1F | TATCGATAAGCTTGCATGCCTGCAGAGATATTTAGACGTGAGACCGGAGCA | 10xPBS(S6) |
| Fluc-S6_2R | CCTATCGCTCGCTGGTGTAAATATCTCATATGAAATATCTTGCTCCGGTCTCACGTCT | 10xPBS(S6) |
| Fluc-S6_3F | ACACCAGCGAGCGATAGGAGATATTTAAGTGTAGATATTTTCCGAGAGCTGGAAGGGA | 10xPBS(S6) |
| Fluc-S6_4R | TGCATGGAGGTTGGCCAGAAATATCTTAAGGAAAATATCTTCCCTTCCAGCTCTCGGA | 10xPBS(S6) |
| Fluc-S6_5F | CTGGCCAACCTCCATGCAAGATATTTAAGGAGAGATATTTGTGGCAGCCAGCATCTCG | 10xPBS(S6) |
| Fluc-S6_6R | GGGGTCACAGGGATGCCACCAAATATCTCGAGATGCTGGCTGCCAC | 10xPBS(S6) |
|  |  |  |
| Fluc-S8_1F | TATCGATAAGCTTGCATGCCTGCAGATATATGTAGACGTGAGACCGGAGCA | 10xPBS(S8) |
| Fluc-S8_2R | CCTATCGCTCGCTGGTGTACATATATCATATGACATATATTGCTCCGGTCTCACGTCT | 10xPBS(S8) |
| Fluc-S8_3F | ACACCAGCGAGCGATAGGATATATGTAAGTGTATATATGTTCCGAGAGCTGGAAGGGA | 10xPBS(S8) |
| Fluc-S8_4R | TGCATGGAGGTTGGCCAGACATATATTAAGGAACATATATTCCCTTCCAGCTCTCGGA | 10xPBS(S8) |
| Fluc-S8_5F | CTGGCCAACCTCCATGCAATATATGTAAGGAGATATATGTGTGGCAGCCAGCATCTCG | 10xPBS(S8) |
| Fluc-S8_6R | GGGGTCACAGGGATGCCACCACATATATCGAGATGCTGGCTGCCAC | 10xPBS(S8) |
|  |  |  |
| Fluc-A_1F | TATCGATAAGCTTGCATGCCTGCAGTGTGTGGAAGACGTGAGACCGGAGCA | 10xPBS(A) |
| Fluc-A_2R | CCTATCGCTCGCTGGTGTTCCACACACATATGTCCACACATGCTCCGGTCTCACGTCT | 10xPBS(A) |
| Fluc-A_3F | ACACCAGCGAGCGATAGGTGTGTGGAAAGTGTTGTGTGGATCCGAGAGCTGGAAGGGA | 10xPBS(A) |
| Fluc-A_4R | TGCATGGAGGTTGGCCAGTCCACACATAAGGATCCACACATCCCTTCCAGCTCTCGGA | 10xPBS(A) |
| Fluc-A_5F | CTGGCCAACCTCCATGCATGTGTGGAAAGGAGTGTGTGGAGTGGCAGCCAGCATCTCG | 10xPBS(A) |
| Fluc-A_6R | GGGGTCACAGGGATGCCACCTCCACACACGAGATGCTGGCTGCCAC | 10xPBS(A) |
|  |  |  |
| Fluc-B_1F | TATCGATAAGCTTGCATGCCTGCAGAGTATAATAGACGTGAGACCGGAGCA | 10xPBS(B) |
| Fluc-B_2R | CCTATCGCTCGCTGGTGTATTATACTCATATGATTATACTTGCTCCGGTCTCACGTCT | 10xPBS(B) |
| Fluc-B_3F | ACACCAGCGAGCGATAGGAGTATAATAAGTGTAGTATAATTCCGAGAGCTGGAAGGGA | 10xPBS(B) |
| Fluc-B_4R | TGCATGGAGGTTGGCCAGATTATACTTAAGGAATTATACTTCCCTTCCAGCTCTCGGA | 10xPBS(B) |
| Fluc-B_5F | CTGGCCAACCTCCATGCAAGTATAATAAGGAGAGTATAATGTGGCAGCCAGCATCTCG | 10xPBS(B) |
| Fluc-B_6R | GGGGTCACAGGGATGCCACCATTATACTCGAGATGCTGGCTGCCAC | 10xPBS(B) |
|  |  |  |
| Fluc-C_1F | TATCGATAAGCTTGCATGCCTGCAGTCTTTAAAAGACGTGAGACCGGAGCA | 10xPBS(C) |
| Fluc-C_2R | CCTATCGCTCGCTGGTGTTTTAAAGACATATGTTTAAAGATGCTCCGGTCTCACGTCT | 10xPBS(C) |
| Fluc-C_3F | ACACCAGCGAGCGATAGGTCTTTAAAAAGTGTTCTTTAAATCCGAGAGCTGGAAGGGA | 10xPBS(C) |
| Fluc-C_4R | TGCATGGAGGTTGGCCAGTTTAAAGATAAGGATTTAAAGATCCCTTCCAGCTCTCGGA | 10xPBS(C) |
| Fluc-C_5F | CTGGCCAACCTCCATGCATCTTTAAAAAGGAGTCTTTAAAGTGGCAGCCAGCATCTCG | 10xPBS(C) |
| Fluc-C_6R | GGGGTCACAGGGATGCCACCTTTAAAGACGAGATGCTGGCTGCCAC | 10xPBS(C) |
|  |  |  |
| Fluc-D_1F | TATCGATAAGCTTGCATGCCTGCAGTGTAATATAGACGTGAGACCGGAGCA | 10xPBS(D) |
| Fluc-D_2R | CCTATCGCTCGCTGGTGTATATTACACATATGATATTACATGCTCCGGTCTCACGTCT | 10xPBS(D) |
| Fluc-D_3F | ACACCAGCGAGCGATAGGTGTAATATAAGTGTTGTAATATTCCGAGAGCTGGAAGGGA | 10xPBS(D) |
| Fluc-D_4R | TGCATGGAGGTTGGCCAGATATTACATAAGGAATATTACATCCCTTCCAGCTCTCGGA | 10xPBS(D) |
| Fluc-D_5F | CTGGCCAACCTCCATGCATGTAATATAAGGAGTGTAATATGTGGCAGCCAGCATCTCG | 10xPBS(D) |
| Fluc-D_6R | GGGGTCACAGGGATGCCACCATATTACACGAGATGCTGGCTGCCAC | 10xPBS(D) |
|  |  |  |
| Fluc-E_1F | TATCGATAAGCTTGCATGCCTGCAGACATTATAAGACGTGAGACCGGAGCA | 10xPBS(E) |
| Fluc-E_2R | CCTATCGCTCGCTGGTGTTATAATGTCATATGTATAATGTTGCTCCGGTCTCACGTCT | 10xPBS(E) |
| Fluc-E_3F | ACACCAGCGAGCGATAGGACATTATAAAGTGTACATTATATCCGAGAGCTGGAAGGGA | 10xPBS(E) |
| Fluc-E_4R | TGCATGGAGGTTGGCCAGTATAATGTTAAGGATATAATGTTCCCTTCCAGCTCTCGGA | 10xPBS(E) |
| Fluc-E_5F | CTGGCCAACCTCCATGCAACATTATAAAGGAGACATTATAGTGGCAGCCAGCATCTCG | 10xPBS(E) |
| Fluc-E_6R | GGGGTCACAGGGATGCCACCTATAATGTCGAGATGCTGGCTGCCAC | 10xPBS(E) |
|  |  |  |

Table S4. Primer list for effector plasmid cloning.

| **Primer name** | **Primer sequence** | **Amplicon** |
| --- | --- | --- |
|  | **GA cloning of pCMV-TTP(WT)-PUM-HD** |  |
| Flag-TTP-F | CCACC ATG GACTACAAGGATGACGACGATAAA ATGGATCTGACTGCCATCTACGAGA | TTP-GS |
| TTP-GS-R | CTGAACCGCCACCTCCGCTTCCGCCACCTCC CTCAGAAACAGAGATGCGATTGAAGATGG | TTP-GS |
| GS-PUM-F | GCGGAGGTGGCGGTTCAGGTGGCGGTGGATCTGGAGGCGGTGGG GGCCGCAGCCGCCTTT | GS-PUM-HD |
| PUM-stop-pCMV-R | CCACCCGGGATCCTCTAGAGTCGAC TTA CCCTAAGTCAACACCGTTCTTCATGT | GS-PUM-HD |
| pAterm-F | GTCGACTCTAGAGGATCCCGGGTGGCATC | 2.2 kb pCMV5 fragment |
| pCMV5-3235-R | CGCTGAGATAGGTGCCTCACTG | 2.2 kb pCMV5 fragment |
| pCMV5-3183-F | AACTTGGTCTGACAGTTACCAATGCTT | 2.4 kb pCMV5 fragment |
| pCMV-896-Flag-R | TTTATCGTCGTCATCCTTGTAGTCCAT GGTGG GCTTATATAGACCTCCCACCGTACA | 2.4 kb pCMV5 fragment |
|  |  |  |
|  | **GA cloning of pCMV-TTP(WT)** |  |
| Flag-TTP-F | CCACC ATG GACTACAAGGATGACGACGATAAA ATGGATCTGACTGCCATCTACGAGA | TTP-stop |
| TTP-stop-pCMV-R | CCACCCGGGATCCTCTAGAGTCGAC TTA CTCAGAAACAGAGATGCGATTGAAGATGG | TTP-stop |
| pAterm-F | GTCGACTCTAGAGGATCCCGGGTGGCATC | 2.2 kb pCMV5 fragment |
| pCMV5-3235-R | CGCTGAGATAGGTGCCTCACTG | 2.2 kb pCMV5 fragment |
| pCMV5-3183-F | AACTTGGTCTGACAGTTACCAATGCTT | 2.4 kb pCMV5 fragment |
| pCMV-896-Flag-R | TTTATCGTCGTCATCCTTGTAGTCCAT GGTGG GCTTATATAGACCTCCCACCGTACA | 2.4 kb pCMV5 fragment |
|  |  |  |
|  | **GA cloning of pCMV-PUM-HD** |  |
| Flag-PUM-F | CCACC ATG GACTACAAGGATGACGACGATAAA GGCCGCAGCCGCCTTT | Flag-PUM-HD |
| PUM-stop-pCMV-R | CCACCCGGGATCCTCTAGAGTCGAC TTA CCCTAAGTCAACACCGTTCTTCATGT | Flag-PUM-HD |
| pAterm-F | GTCGACTCTAGAGGATCCCGGGTGGCATC | 2.2 kb pCMV5 fragment |
| pCMV5-3235-R | CGCTGAGATAGGTGCCTCACTG | 2.2 kb pCMV5 fragment |
| pCMV5-3183-F | AACTTGGTCTGACAGTTACCAATGCTT | 2.4 kb pCMV5 fragment |
| pCMV-896-Flag-R | TTTATCGTCGTCATCCTTGTAGTCCAT GGTGG GCTTATATAGACCTCCCACCGTACA | 2.4 kb pCMV5 fragment |
|  |  |  |
